# Comprehensive Pharmacobotanical and Phytochemical Profiling of *Glechoma hederacea* L. from Bihor County, North-West Romania

**DOI:** 10.3390/life15091466

**Published:** 2025-09-18

**Authors:** Manuela Bianca Pasca, Alicia-Denisa Costa, Daniela Gitea, Corina Moisa, Tunde Jurca, Cristina Burlou-Nagy (Fati), Neli Kinga Olah, Annamaria Pallag, Manuel Alexandru Gitea

**Affiliations:** 1 Department of Pharmacy, Faculty of Medicine and Pharmacy, University of Oradea, 410028 Oradea, Romania; bpasca@uoradea.ro (M.B.P.); tjurca@uoradea.ro (T.J.); burlounagy.cristina@student.uoradea.ro (C.B.-N.); apallag@uoradea.ro (A.P.); 2 Doctoral School of Biological and Biomedical Sciences, University of Oradea, 410087 Oradea, Romania; costa.aliciadenisa@student.uoradea.ro; 3 Department of Pharmaceutical Chemistry, Faculty of Pharmacy, Vasile Goldis Western University of Arad, 310414 Arad, Romania; neliolah@yahoo.com; 4 Department of Agriculture, Horticulture, Faculty of Environmental Protection, University of Oradea, 410048 Oradea, Romania; mgitea@uoradea.ro

**Keywords:** *Glechoma hederacea* L., histo-anatomical characters, optical microscopy, phytochemical, HPLC analysis, antioxidant capacity

## Abstract

*Glechoma hederacea* L. (GH) is an understudied species with significant phytotherapeutic potential, whose botanical characteristics and phytochemical profile have not previously been investigated from Bihor County, North-West Romania, namely Săldăbagiu de Munte (261 m a.s.l.) and Beiuș (553 m a.s.l.). In this study, we aimed to examine the species from both pharmacobotanical and phytochemical perspectives focusing on two populations originating from areas with different altitudes, which were selected as representative sites for collecting GH populations. The morphological analysis included both macroscopic and microscopic examinations performed with optical microscopy, complemented by phytochemical profiling and antioxidant activity evaluation. The phenolic profile was analyzed by high-performance liquid chromatography. Total phenolic content was determined using the Folin–Ciocalteu method, while total flavonoid content was assessed by the aluminum chloride colorimetric assay. In addition, the total anthocyanin content was determined, too. The antioxidant properties of the samples were evaluated using cupric ion reducing antioxidant capacity (CUPRAC) and ferric reducing antioxidant power (FRAP) assays. Our results indicate that GH from the higher-altitude area is a rich source of phenolics and exhibits notable antioxidant activity. Among the identified active compounds, apigenin and caffeic acid were found in the highest concentrations. These findings support the potential use of this species in phytopharmaceutical formulations.

## 1. Introduction

The *Glechoma* L. genus belongs to the *Lamiaceae* family and includes eight perennial species distributed across Europe and Asia, with the center of diversity in China. *Glechoma hederacea* L. is the most widespread species of the genus [1].

In Romania, the species *Glechoma hederacea* L. (GH) and *Glechoma hirsuta* Waldst. et Kit. are present in the wild flora [2,3,4,5]. Of these two species, GH is the most common in Romania. It grows in areas with high soil moisture, in forests, pastures, and scrubland in the Codru-Moma Mountains, the Semenic Mountains, and the Zarand Mountains; as well as in deciduous forests near Cluj, Bihor, Bucharest, Bran and Prahova [3,6]. In north-west Romania, GH is locally known as *rotunjioară*, *buruiana zgaibii*, or *silnic* [6,7,8]. Commonly referred to as ground ivy in English. GH is a perennial plant which has a series of morphological and anatomical characteristics specific to the genus *Glechoma* and the Lamiaceae family in general. Species from Lamiaceae family are characterized by tetragonal stems, opposite leaves, verticillate inflorescences, and the presence of protective and glandular trichomes associated with the secretion of essential oils. The genus *Glechoma* is characterized by creeping stems, reniform to cordate leaves with wavy margins, and purple bilabiate corollas arranged in axillary cymes. Anatomically, GH has angular collenchyma in the corners of the stem, an adaxial epidermis covered with multicellular protective trichomes and an abaxial epidermis. The leaves have secretory glands, which is characteristic for the Lamiaceae family.

**Figure 1:**
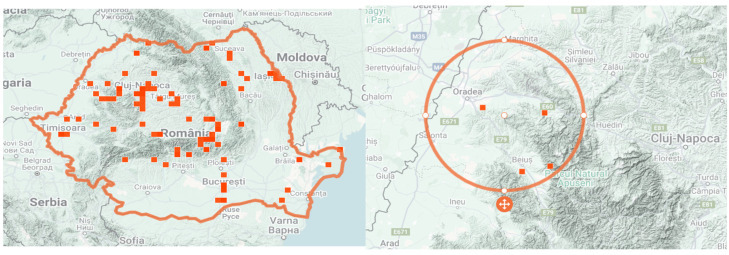
Geographical distribution of *Glechoma hederacea* L. in Bihor County (NW Romania), marked online on iNaturalist [9].

Figure 1 illustrates the geographical distribution of GH in Bihor County (NW Romania) based on validated occurrence records from the iNaturalist platform, which was consulted for species confirmation within Romania.

Although it is not well-known as a medicinal plant in the specialized literature and its therapeutic effects have been insufficiently studied, GH has been used for generation in traditional medicine in north-western Romania since the time of the Dacians for the following properties: anti-inflammatory, expectorant, antipyretic, antidiarrheal, diuretic, vermifuge, analgesic, antiscorbutic, disinfectant, antispasmodic, healing, and emollient [6,10,11,12]. The freshly harvested leaves have been used to prepare topical remedies (infusions, tinctures, ointments) for the treatment of various conditions like eczema, burns, abscesses, contusions, wounds, boils, or anthrax-specific lesions (known as *buba vânătă*) [13,14]. GH has also been used in veterinary medicine as a vermifuge [6].

In allopathic medicine, GH can be used as an immunotherapeutic agent to combat certain types of inflammation, as it is very effective in stopping the inflammatory process [15,16]. The aerial parts of the GH are rich in various active compounds such as: flavonoids (quercetin, luteolin, apigenin), essential oils (rich in camphene, pinocamphene, pinene, menthone, myrcene, pulegone), pentacyclic triterpenic acids (ursolic acid, 2-α-hydroxyursolic acid, 2-β-hydroxyursolic acid, asiatic acid), sesquiterpenoids (glechomanolide, glechomafuran), phenolic acids (rosmarinic acid, ferulic acid, caffeic acid, syringic acid, vanillic acid), fatty acids, bitter principles, saponosides, tannins (6–7 buckthorn) [17,18,19,20,21]. Additionally, the mineral content varies based on the harvesting region and may contain trace amounts of arsenic, barium, bromine, chlorine, chromium, copper, iodine, iron, phosphorus, manganese, molybdenum, nickel, potassium, zinc, and titanium [22]. Rosmarinic acid is one of the most abundant plant esters formed from caffeic acid and 3,4-dihydroxyphenyl lactic acid, and is present in high concentrations in GH [16,23].

The interest of scientists in the study of GH is due to the presence of a large number of bioactive compounds. Thanks to the reducing effect of its constituents, GH has been used for the biological synthesis of Au/ZnO/Ag nanoparticles that exhibit cytotoxic activity against leukemia [19]. Another study has shown that the GH extract inhibits melanin synthesis in B16 melanoma cells without having a cytotoxic effect. Thus, GH could be useful in treating hyperpigmentation and skin whitening [24]. Its antioxidant, antimicrobial and anticancer effects have also been studied, all using in vitro methods [25]. Currently, pharmaceutical research is focused on the discovery of plant species from wild flora, the identification of biologically active compounds and their localization in tissues [26,27].

Environmental conditions play a crucial role in shaping both the morphological traits and the phytochemical composition of medicinal plants. Variations in altitude, temperature, solar radiation, soil properties, and humidity can modulate plant metabolism, often leading to significant differences in the biosynthesis of secondary metabolites such as flavonoids, polyphenols, and anthocyanins. In higher-altitude environments, cooler temperatures and increased UV radiation may stimulate the accumulation of phenolic compounds as part of the plant’s protective mechanisms. Differences in soil pH, nutrient availability, and water retention can further influence growth and metabolic pathways [28,29,30].

In this context, the two GH populations examined in this study—originating from Săldăbagiu de Munte (261 m a.s.l.) and Beiuș (553 m a.s.l.) in Bihor County, Romania—offer an opportunity to investigate how contrasting habitats may influence their morphological development, phytochemical profile, and antioxidant activity. Our research, which aimed to describe the comparative botanical, morpho-anatomical and phytochemical characteristics of the GH1 and GH2 populations, aims not only to document interpopulation differences, but also to highlight ecological adaptations and correlate them with the accumulation of bioactive compounds, which may suggest distinct chemotypes. This information is essential for the correct identification of the plant and represents an important premise for future studies in medicine and pharmacy. To achieve this, we performed macroscopic, microscopic and histochemical analyses of the species, along with the quantitative determination of its components using advanced analytical methods.

## 2. Materials and Methods

### 2.1. Plant Material

For this study, several specimens of GH were identified and collected from the wild flora in unpolluted areas, originating from two regions with different altitudes. The studied populations were from Bihor County in north-west Romania, GH1, from the forest near the village of Săldăbagiu de Munte (47°5′22″ N 21°58′31″ E) at an altitude of 261 m, and GH2, near the town of Beiuș (46°34′47″ N 22°12′59″ E) at an altitude of 553 m. Ten specimens were collected from each location, during the flowering period (between March and May 2024). Healthy specimens were selected, without yellowed, broken, or dried leaves and without signs of parasite infestation. All vegetative organs of the GH, (roots, stems, leaves, and flowers) were examined both macroscopically and microscopically and then subjected to phytochemical analysis. For morpho-anatomical analyses, whole plants were used (approximately 30 g fresh weight per specimen), while for phytochemical determinations, the pooled dried material amounted to ~150 g per population. The codification of the samples, together with their origin and environmental characteristics, is presented in Table 1 [31].

A sample of the species from both populations has been preserved inside the herbarium of the Faculty of Medicine and Pharmacy, University of Oradea, Romania, registered in NYBG Steere Herbarium, UOP 05732 for the GH1 population and UOP 05733 for the GH2 population.

### 2.2. Reagents and Materials

Trolox (6-hydroxy-2,5,5,7,8-tetramethylchroman-2-carboxylic acid), 2,4,6-Tris(2-pyridyl)-S-triazine (TPTZ) and neocuproine (2,9-dimethyl-1, 10-phenanthroline) were purchased from Sigma Aldrich, St. Louis, MO, in the United States. Folin–Ciocalteu reagent, iron (III) chloride hexahydrate (FeCl_3_∙6H_2_O), copper (II) chloride (CuCl_2_), and sodium carbonate (Na_2_CO_3_) were purchased from Carl Roth GmbH + Co KG, Karlsruhe, Germany. Ethanol was purchased from Chimreactiv SRL, Bucharest, Romania. HPLC-grade acetonitrile was purchased from Merck (Darmstadt, Germany) and ultrapure water was purified with the Direct-Q UV system from Millipore (Burlington, MA, USA). Standard chlorogenic and gallic acid (98% HPLC purity), apigenin (99% HPLC purity) were from Sigma (St. Louis, MO, USA).

### 2.3. Macroscopic, Microscopic and Morphological Characterization

The macroscopic characterization of the specimens from the GH1 and GH2 populations was carried out in accordance with the recommendations of the 10th edition of the Romanian Pharmacopoeia by observing the organoleptic characteristics: appearance (size, color), smell and taste for each plant part separately [32]. The plant material was photographed using a Canon EOS R5 camera with a Canon RF 35 mm F1.8 Macro IS STM lens, (Canon Inc., Tokyo, Japan), monocular.

Microscopic preparations were obtained from fresh plant material by making cross-sections of all plant organs, which were then stained with a hydroalcoholic solution of Genevez reagent (Congo red and chrysoidine). The sections were immersed in the staining solution for 5 min and subsequently rinsed several times with distilled water to remove excess dye. Observations and image acquisition were performed using an optical microscope (Optika, model C-B10+ 24010, Ponteranica, Italy) equipped with 10×, 20×, and 40× objectives and an Optika B10 digital camera [32,33].

### 2.4. Phytochemical Analysis

For the phytochemical analyses, we used the whole plant of GH (roots, stems, leaves, and flowers), which were harvested during the flowering stage, dried at an average temperature of 40 °C for 96 h and processed into powdered material for extraction. The resulting dried material was used for the determination of anthocyanin content and for HPLC analysis. For the determination of total polyphenols, total flavonoids, and antioxidant activity, a 10% (*w*/*v*) extract was prepared by macerating the powdered dry plant material in 70% ethanol for 3 days at room temperature, protected from light. After maceration, the mixture was filtered, and the obtained extract was stored at 4 °C until analysis.

#### 2.4.1. Evaluation of Total Phenolic Content (TPC)

Total phenolic content was quantified using the Folin–Ciocalteu assay. Under alkaline conditions provided by sodium carbonate, phenolic hydroxyl groups react with the reagent, producing a measurable color change. The absorbance at 765 nm correlates with the concentration of polyphenolic hydroxyl groups in the sample. The extract solution (0.1 mL) containing 1000 μg of the extract was mixed with 46 mL distilled water in a volumetric flask and 1 mL Folin–Ciocalteu chemical agent was included, and the flask was thoroughly shaken. The blend was permitted to respond for 3 min and 3 mL watery arrangement of 2% Na_2_CO_3_ was included. The gallic acid concentrations used for calibration were 20–100 PPM. At the conclusion of the 2 h hatching at the room temperature, the absorbance of each blend was measured at 765 nm in Shimadzu UV-1700 Pharmaspec UV-VIS Spectrophotometer, Kyoto, Japan [34]. The same strategy was moreover applied to the standard solutions of gallic acid, and a calibration curve was obtained (y = 0.0135x + 0.0832, R^2^ = 0.9963) [35]. TPC of the samples was reported as milligrams of gallic acid (GA) per gram of dry plant material. Each determination was performed in triplicate, and mean values were calculated.

#### 2.4.2. Evaluation of Total Flavonoid Content (TFC)

Total flavonoids were measured by applying a colorimetric procedure described in earlier studies. A 10 mL volumetric flask was filled with a 1 mL alcoholic extract after diluting it with 4 mL of water. The first addition was 3 mL of 5% NaNO_2_ solution, followed by 0.3 mL of 10% AlCl_3_ after 5 min and 2 mL of 1M NaOH after 6 min. Distilled water was added to the flask until it reached the calibration point. Then, the solution was blended, and the Shimadzu UV-1700 Pharmaspec UV-VIS Spectrophotometer, Kyoto, Japan, measured the solution’s absorbance at 510 nm. The calibration curve was obtained using quercetin (Q) standards in the range 0.00–0.10 mg/mL, yielding the equation y = 0.8598x − 0.00015 with R^2^ = 0.9986, TFC was expressed as mg Q/g dw.

#### 2.4.3. Determination of Total Anthocyanin Content

The total anthocyanin content was determined using the pH differential spectrophotometric method, which is based on the property of anthocyanins to change color depending on the pH. At pH 1.0, the oxonium (colored) form predominates, whereas at pH 4.5, the chalcone (colorless) form is present, and the difference in absorption spectra allows accurate quantification even in the presence of polymerized pigments or other interfering compounds. Approximately 0.15 g of powdered dry plant material from each of the two GH populations was homogenized for 1 min at 3000 rpm using a high-speed homogenizer Ultraturrax (IKA T25, IKA^®^-Werke GmbH & Co. KG, Staufen, Germany) in methanol acidified with 0.3% HCl. The mixture was centrifuged at 2404× *g* for 20 min using a laboratory centrifuge (Hettich Universal 320, Andreas Hettich GmbH & Co. KG, Tuttlingen, Germany), and the supernatant was collected. The residue was re-homogenized and centrifuged twice more under the same conditions, and all supernatants were combined to obtain the final extract, which was used for both anthocyanin content determination and antioxidant activity assessment. Two dilutions of the extract were prepared, one in potassium chloride buffer (0.025 M, pH 1.0) and one in sodium acetate buffer (0.4 M, pH 4.5), adjusted so that the absorbance at λ_vis-max did not exceed 1.2. After 15 min at room temperature, absorbance was measured at λ_vis-max and 700 nm against the corresponding buffers [36].

The absorbance (A) difference was calculated as Equation (1) describes:

(1) $$A={\left(A_{\lambda_{vis-max}}-A_{700}\right)}_{pH1.0}-{\left(A_{\lambda_{vis-max}}-A_{700}\right)}_{pH4.5}$$

For the calculation of the monomeric anthocyanin pigment in the sample, the formula used was as follows:

(2) $$\text{The monomeric anthocyanin}\left(\frac{mg}{L}\right)=\frac{A\times Mw\times DF\times 1000}{\varepsilon \times 1}$$

where Mw = 449.2 g/mol for cyanidin-3-glucoside, DF = dilution factor, and ε = 26,900 L·mol^−1^·cm^−1^. The results were expressed as mg cyanidin-3-glucoside equivalents per 100 g dry weight (mg C3G/100 g dw), l = 1 cm.

#### 2.4.4. Characterization of Individual Phenolic Compounds Using HPLC

For extraction, 1 g of sample was mixed with 5 mL of methanol and sonicated for 60 min. The mixture was centrifuged at 15,269× *g* for 10 min, after which the supernatants were collected, microfiltered as previously described, and subjected to HPLC with diode array detection (DAD). Analyses were performed on a Shimadzu Nexera-I HPLC system equipped with a Fortis C18 silica column (150 × 2.1 mm, 3 µm), using an acidified water–acetonitrile gradient, as detailed below. The mobile phase was a linear gradient using water with 0.1% formic acid at pH 2.5 and acetonitrile. The column used for HPLC analysis is Fortis C18, 150 × 2.1 mm × 3 µm manufactured by Fortis Technologies Ltd., Cheshire, UK. The column was maintained at 40 °C during the separation and from each sample and standard were injected 10 μL. The used flow rate was 1 mL/min. For each standard a calibration curve was built and the curve equation, LOD and LOQ were established. A linear gradient elution was applied, starting at 80% formic acid, decreasing to 60% over 5 min, to 40% at 10 min, and to 20% at 15 min, where it was maintained for 5 min. The solvent concentration was then reduced to 10% and held for another 5 min, followed by a gradual increase to 20% over the next 5 min, and finally rose progressively back to 80% at the end of the 40 min run. Spectral data were recorded across 220–600 nm. Linearity of the detector response was verified using apigenin and caffeic acid standards [37]. Results were reported as mg per 100 g dry weight (dw).

### 2.5. Determination of Antioxidant Activity by FRAP and CUPRAC Methods

The antioxidant activity of the alcoholic extract obtained from the whole plant of two GH populations harvested from different geographical areas was determined by three complementary methods: FRAP (Ferric Reducing Antioxidant Power) and CUPRAC (Cupric Ion Reducing Antioxidant Capacity).

The FRAP assay measures antioxidant capacity based on the reduction of the Fe(III)-TPTZ complex to Fe(II)-TPTZ under acidic conditions, monitored spectrophotometrically. The stock solutions were prepared as follows: 270 mg of FeCl_3_·6H_2_O dissolved in 50 mL distilled water; 50 mg of TPTZ with 1 mL HCl dissolved in 50 mL distilled water; and 300 mM acetate buffer adjusted to pH 4.5. The FRAP working solution was freshly prepared by mixing 5 mL of FeCl_3_·6H_2_O solution, 5 mL of TPTZ solution, and 50 mL of acetate buffer, followed by incubation for 60 min. Trolox served as the reference standard, and a calibration curve was established in the range of 0–300 μM, with a correlation coefficient of R^2^ = 0.9956 and the regression equation y = 0.0017x + 0.0848, where y denotes the absorbance measured at 595 nm [27,38]. Results are expressed as μmol Trolox equivalents (TE) per g of dry weight (dw).

The antioxidant reducing capacity of cupric ions was determined using the CUPRAC method [39], with minor modifications.

The process consisted of placing 0.25 mL ethanolic solution of neo-cuproine (7.5 × 10^−3^ M), 0.25 mL cupric chloride solution (0.01 M) and 0.25 mL ammonium acetate buffer solution (1 M) in a test tube and mixing them with the plant extract. The entire amount is mixed gently and adjusted with distilled water to 2 mL then, stoppering the tubes, left at room temperature for 30 min. Trolox was used as the reference standard, and a calibration curve was constructed for concentrations ranging from 0 to 2500 μM. After 30 min of incubation, absorbance was recorded at 450 nm against a reagent blank. An increase in absorbance indicated a higher reducing capacity of the sample [4,28]. The measuring units for the obtained results was μmol Trolox equivalent (TE)/g dw.

### 2.6. Statistical Analysis

All measurements were carried out in triplicate, and results are presented as mean ± standard deviation (SD). Statistical analyses were performed using JASP software, version 0.18 (JASP Team, Amsterdam, The Netherlands), with independent samples *t*-tests applied to compare the means of the two populations (GH1 and GH2). Differences were considered statistically significant at *p* < 0.05. In tables, different letters within the same column indicate significant differences between samples.

## 3. Results

### 3.1. Macroscopic Characterization

**Figure 2:**
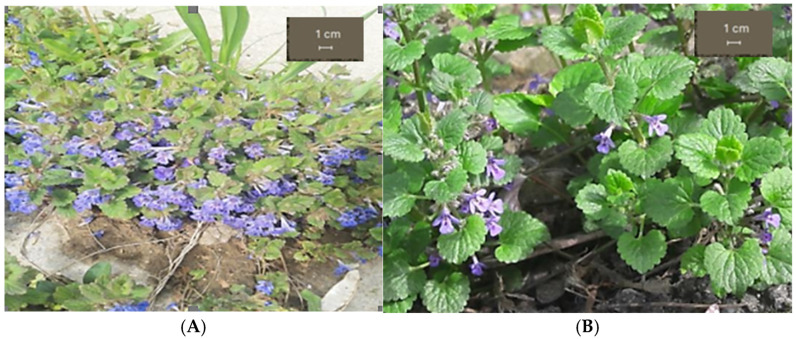
*Glechoma hederacea* L. in its natural habitat; (**A**) GH1; (**B**) GH2.

The macroscopic analysis was carried out comparing the GH1 and the GH2 populations (Figure 2). The morphological differences in their vegetative parts are due to altitude, soil type and light exposure.

**Figure 3:**
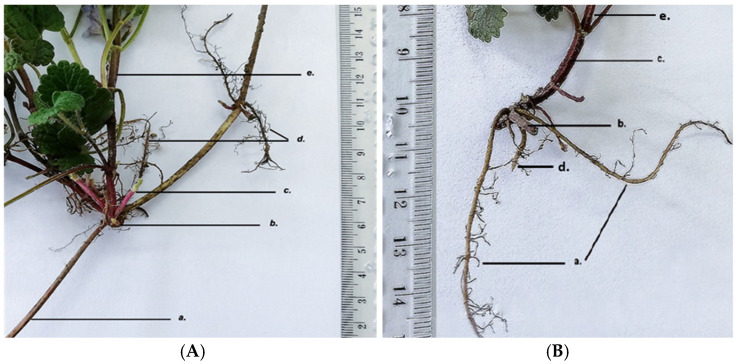
*Glechoma hederacea* L. root; (**A**) GH1; (**B**) GH2; a. plagiotropic stem; b. rhizome; c. young shoots; d. root; e. aerial stem.

GH has a fasciculate monopodial root system with order II and order III roots that serve to anchor the plant in the soil and to absorb nutrients and water. The species has plagiotropic or creeping stems (Figure 3).

**Figure 4:**
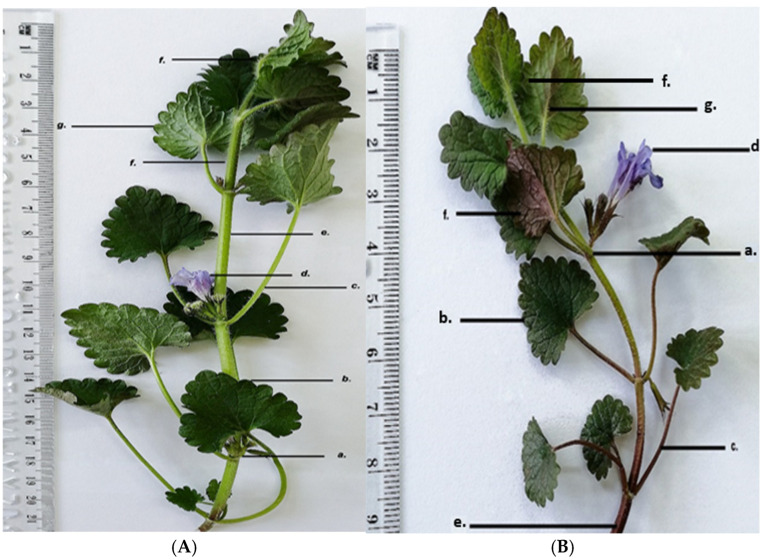
*Glechoma hederacea* L. herba; (**A**) GH1; (**B**) GH2; a. node; b. leaf—adaxial face; c. petiole; d. flower; e. aerial stem; f. trichomes; g. leaf—abaxial face.

GH has the following morphological characteristics on its aerial parts: the stem is orthotropic, herbaceous, four-sided; the petiole is oppositely inserted at the nodes; the leaves are reniform with crenate margins; the flowers are arranged in cymes, inserted at the nodes; the entire aerial surface is covered with protective trichomes that are unevenly distributed over the organs. Morphologically, the two harvested populations of GH show the same structural elements, with some morphological differences as shown in Figure 4.

GH1 plants are taller (21–22 cm) with a light green, four-sided stem 6–7 mm thick, and a petiole of 4–5 cm, longer than the leaf blade (~3 cm). The lamina has a dark green adaxial surface and a lighter, slightly greyish abaxial surface; flowers are light purple.

GH2 plants are shorter (10–11 cm), with a four-sided stem 5–6 mm thick, green with purple tinges. The petiole (2–2.5 cm), equal in length to the leaf blade, is green with violet tinges. The lamina has a dark green adaxial surface and an abaxial surface with brown-purple shades; flowers are a darker purple, making them more visually striking.

**Figure 5:**
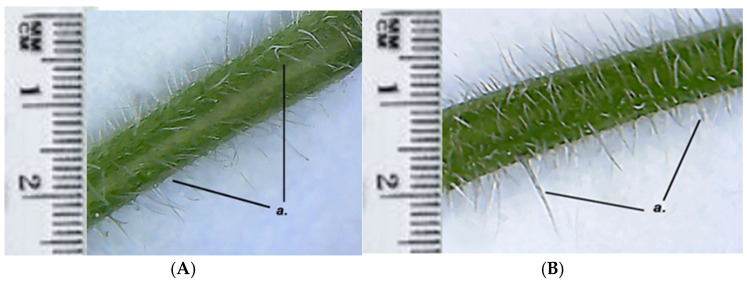
*Glechoma hederacea* L. stem; (**A**) GH1; (**B**) GH2; a. multicellular protective trichomes.

**Figure 6:**
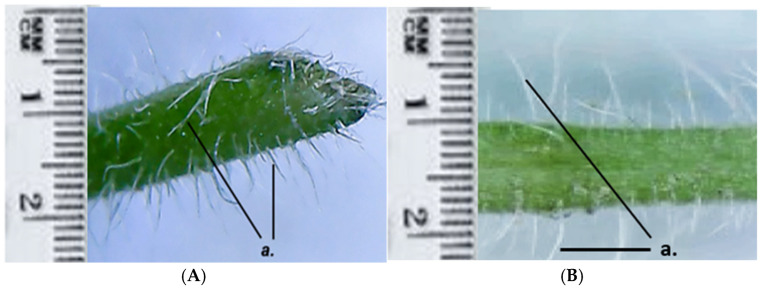
*Glechoma hederacea* L. petiole; (**A**) GH1; (**B**) GH2; a. protective trichomes.

Both GH1 and GH2 have multicellular protective trichomes of different lengths on the stem. Compared to GH1, GH2 has a higher density of protective trichomes per cm^2^, which are also longer and thicker (Figure 5). The same differentiation was observed on the petiole; GH2 has a greater number of protective trichomes, which are longer and thicker compared to those of GH1 (Figure 6).

**Figure 7:**
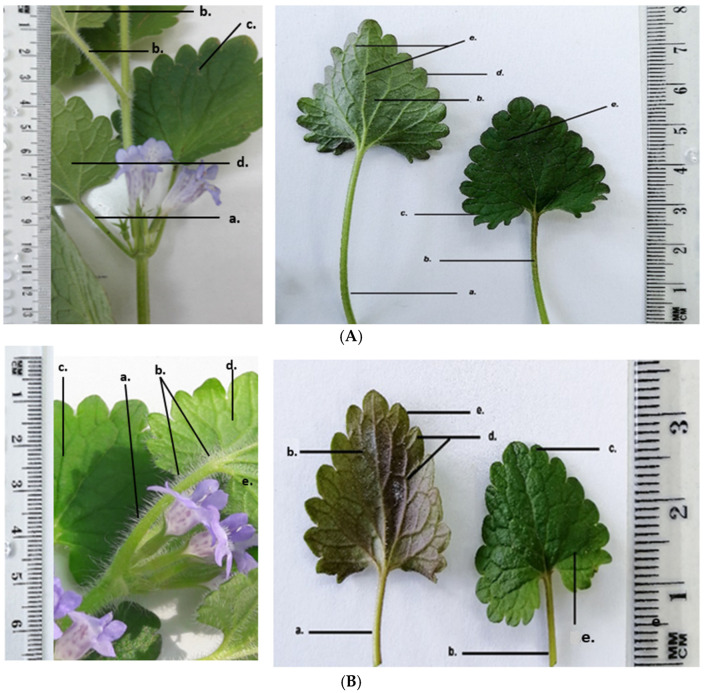
*Glechoma hederacea* L. leaf; (**A**) GH1; (**B**) GH2: a. petiole; b. protective trichomes; c. adaxial face; d. abaxial face; e. veins.

**Figure 8:**
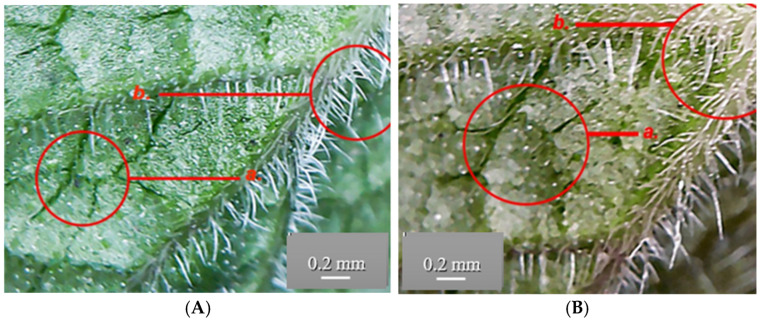
*Glechoma hederacea* L. leaf—abaxial face; (**A**) GH1; (**B**) GH2: a. veins; b. protective trichomes.

Both the GH1 and the GH2 populations have reniform leaves with crenate margins, arranged opposite one another, and with a clearly visible palmate venation on both surfaces. On the adaxial face, the veins are indented into the leaf blade, while on the abaxial surface they are prominent and raised, giving a velvety texture of the leaf. Differences were observed between the leaves of GH1 and GH2 especially on the abaxial and adaxial surfaces of the lamina (Figure 7 and Figure 8). The leaves of GH1 have a dark green adaxial surface and a lighter, slightly greyish abaxial surface, with veins of the same color as the lamina. The petiole is 4–5 cm long, longer than the blade (~3 cm), and of similar color to the stem. In contrast, GH2 leaves show a dark green adaxial surface and an abaxial surface with brown areas and purple shades. Their petiole is brown, shorter (2–2.5 cm), and equal in length to the leaf blade. In both populations, the leaves are densely covered with protective trichomes on both surfaces. The veins on the abaxial surface are light brown. The shape of the leaves differs slightly between the two populations. In GH1, the lamina appears slightly elongated and the reniform curvature at the base of the leaf blade is more pronounced.

In both GH populations, the distribution and density of the protective trichomes on the leaf around the main veins are significantly higher. The GH2 leaves are significantly longer and contain many more protective trichomes than GH1 leaves (Figure 8).

**Figure 9:**
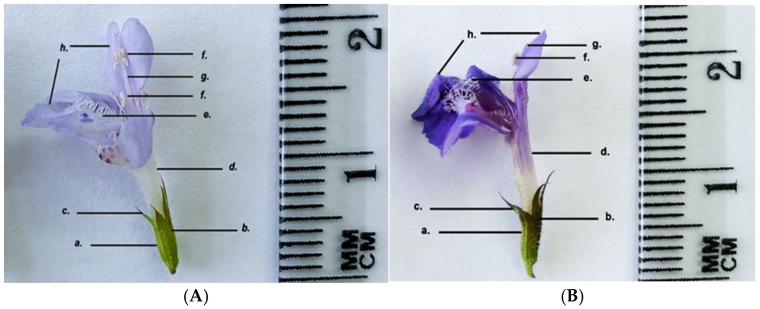
*Glechoma hederacea* L. flower; (**A**) GH1; (**B**) GH2: a. calyx; b. trichomes; c. sepals; d. gamopetalous corolla; e. protective trichomes; f. stamens; g. style; h. petals.

**Figure 10:**
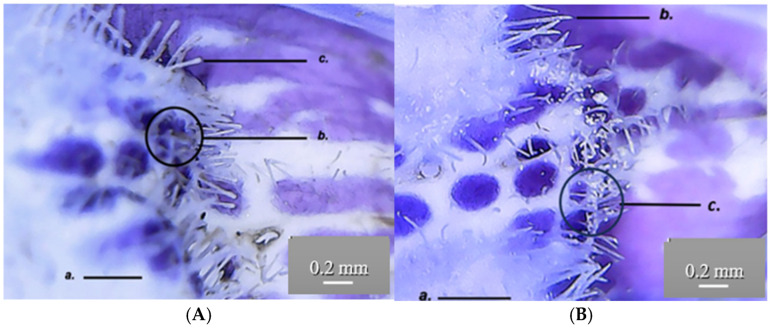
*Glechoma hederacea* L. petal; (**A**) GH1; (**B**) GH2; a. petal; b. pollen; c. protective trichomes.

Both the GH1 and GH2 populations have zygomorphic, dipetalous flowers that are arranged in cymes consisting of 5–6 flowers. The calyx is foliaceous, gamosepalous, infundibuliform, persistent, with five sepals. The sepals are green, with brown-purple tinges in GH2. The corolla is concrescent, bilabiate, light purple in GH1, and dark purple in GH2. Two of the upper petals form the upper lip, while the remaining petals form the lower lip, being covered with numerous protective trichomes (Figure 9 and Figure 10).

The shape of the trichomes on the petals is different in populations GH1 and GH2. In GH1, the trichomes on the lower lip are thicker and more rounded at the tip than in GH2; however, the density of these trichomes is the same in both populations. This could be a polymorphism within the two populations. Regarding the coloration of the petals, the upper petals (upper lip) are uniformly colored in shades of purple, while the lower petals have dark purple spots or insertions. Both the GH1 and GH2 populations have a didynamous androecium and an inferior, tricarpellary gynoecium. The stigma of the gynoecium is bifurcated.

**Figure 11:**
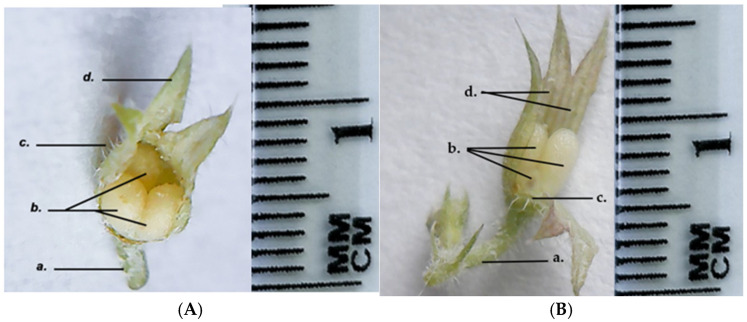
*Glechoma hederacea* L. fruit; (**A**) GH1; (**B**) GH2; a. peduncle; b. fruit (nutlet); c. protective trichomes; d. persistent sepals.

The fruit of the GH species is simple, dry, indehiscent, multi-seeded capsules composed of three nutlets, in contrast to most species of the Lamiaceae Family that usually produce four one-seeded nutlets. The sepals remain persistent throughout the fruit development (Figure 11).

A comparative overview of the main morphological characteristics of GH1 and GH2 is provided in Table 2 (mean ± SD).

### 3.2. Microscopic Characterization

**Figure 12:**
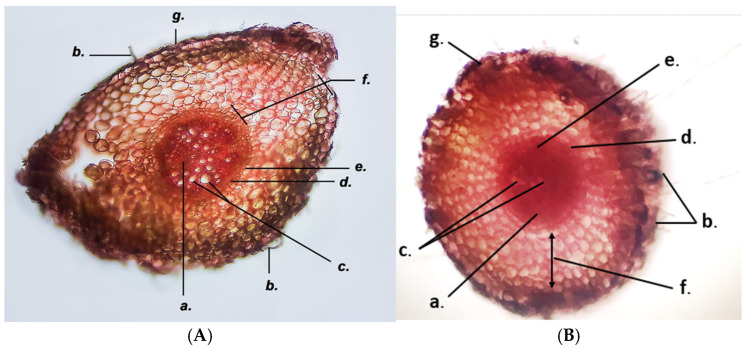
Cross section through *Glechoma hederacea* L. root (10×); stained with Genevez reagent, (**A**) GH1; (**B**) GH2; a. phloem; b. absorptive trichomes; c. xylem; d. pericycle; e. endodermis; f. cortex; g. exodermis.

The GH root has an oval shape in cross-section and consists of the following parts, illustrated in Figure 12: the exodermis (g) is unstratified, with thin cellulose walls and tightly connected cells; the cortex (f) is a pluristratified fundamental tissue, also known as the cortical parenchyma (mesoderm) and has an oval shape, intercellular spaces and thin cellulose walls; the endodermis (e) is the innermost layer of the cortex, consisting of tightly packed, orderly arranged cells whose walls are thickened with lignin; the pericycle (d) is the next layer of cells adjacent to the endodermis and the central cylinder, with thin cellulose walls; the central cylinder shows an alternating arrangement of phloem and xylem conducting vessels and is strongly colored in red, especially in the area of the phloem tissue (a). The distinction between protophloem and metaphloem is not evident. The xylem bundles (c) are divided into xylem vessels which form the protoxylem (located near the pericycle, with smaller diameter) and the metaxylem (located towards the center of the root, with larger diameter) (Figure 12).

**Figure 13:**
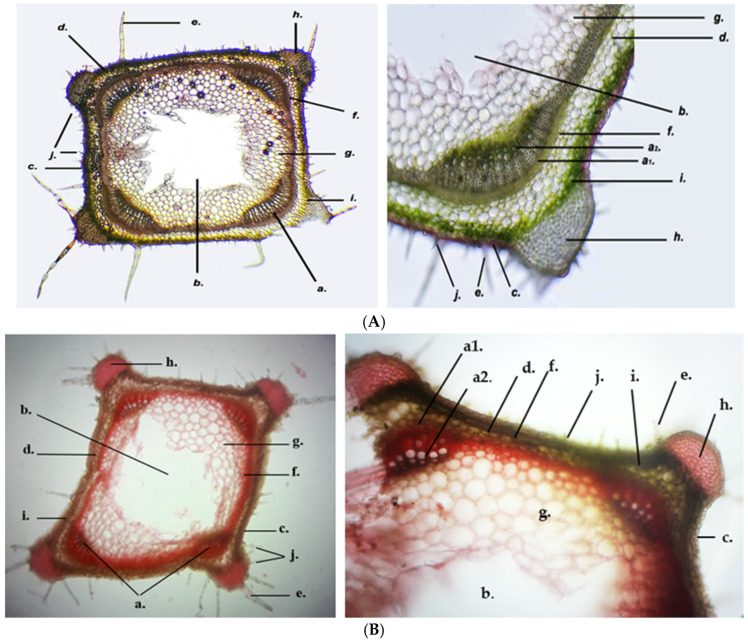
Cross section through *Glechoma hederacea* L. stem (10×; 40×), stained with Genevez reagent, (**A**) GH1; (**B**) GH2; a. vascular (phloem and xylem) tissue; a1. primary phloem tissue; a2. primary xylem tissue; b. central lacuna; c. epidermis; d. cortex; e. mature multicellular trichomes; f. pericycle; g. medullary parenchyma tissue; h. angular collenchyma; i. assimilating parenchyma tissue; j. immature multicellular trichomes.

**Figure 14:**
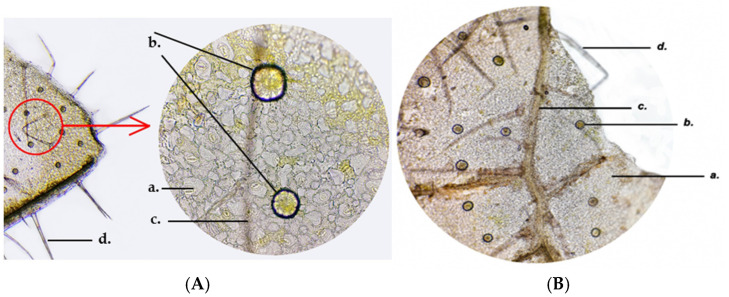
*Glechoma hederacea* L. leaf—abaxial epidermis, secretory glands (10×); (**A**) GH1; (**B**) GH2; a. stomata; b. secretory gland; c. vein, vascular (phloem and xylem) tissue; d. protective trichomes.

In cross-section, the GH stem has a tetragonal shape and exhibits several types of tissue, as follows in Figure 13 and Figure 14. The epidermis (c) is unstratified and appears pink in color near the angular collenchyma (h). This is due to the presence of anthocyanins and the absence of chloroplasts. The epidermal cells are tightly packed, with slightly bulging outer walls. The epidermis has mature (e) and immature (j) multicellular trichomes. At the four corners of the stem is the angular collenchyma (h), characteristic of developing plants, the cell walls are strongly thickened with pectin and cellulose. The assimilating parenchyma tissue (i) lies beneath the epidermis and consists of 2–3 layers of cylindrical cells orientated perpendicular to the epidermal surface. These cells are rich in chloroplasts and play a role in photosynthesis. The cortex (d) consists of three layers of cells with intercellular spaces. The number of chloroplasts in these cells is significantly lower compared to those in the assimilating parenchyma tissue. The pericycle (f) is the next cell layer directly adjacent to the cortex and central cylinder. The cells have thin cellulose walls. GH has mixed vascular bundles (a) at the four corners of the stem, opposite the angular collenchyma. The xylem consists of the protoxylem (smaller diameter) and the metaxylem (larger diameter, located towards the center). The phloem tissue consists of still-living cells and is differentiated into protophloem and meta phloem. The medullary parenchyma tissue (g) consists of several layers of large cells with thin cellulose walls. The central lacuna (b) can be seen in the center (Figure 13).

The anatomical structure of the GH leaf includes the following parts: the adaxial epidermis, the assimilating parenchyma, differentiated into palisade parenchyma and spongy parenchyma, and the abaxial epidermis. The surface of the epidermis has various structures: stomata, secretory glands, and multicellular protective trichomes. The stomata originate from the epidermal cells and play a primary role in gas exchange between the plant and the external environment. Stomata are abundant on both the adaxial and abaxial surfaces and consist of two kidney-shaped stomatal cells facing each other and filled with chloroplasts. Between the stomatal cells lies the ostiole, the opening that enables gas exchange. The subsidiary cells determine the stomatal type, in this case, the diacytic cells.

The secretory glands are seen on the epidermis, each consisting of eight secretory cells tightly joined together (Figure 14).

**Figure 15:**
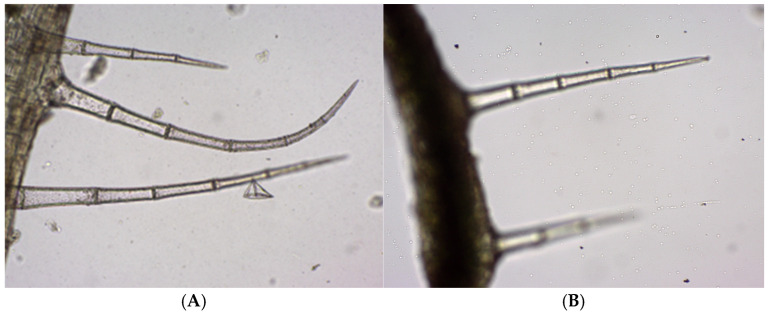
*Glechoma hederacea* L. leaf—protective trichomes (40×); (**A**) GH1; (**B**) GH2.

Trichomes are appendages developed from epidermal cells that elongate and divide perpendicularly to the epidermis. The epidermis bears multicellular protective unbranched trichomes distributed along the leaf, stem and petal surfaces (Figure 15).

**Figure 16:**
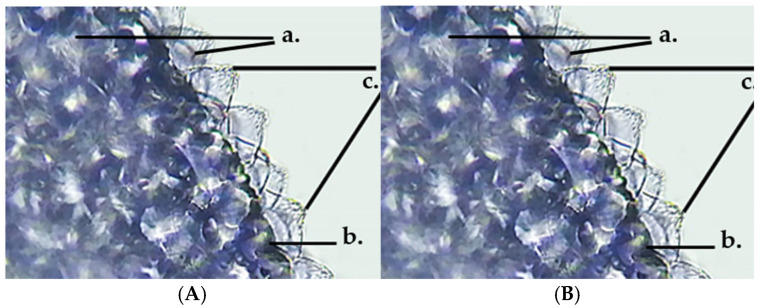
*Glechoma hederacea* L. petals (40×)—secretory papillae; (**A**) GH1; (**B**) GH2; a. anthocyanin accumulated in vacuoles; b. unstratified epidermis; c. secretory papillae.

At the level of the GH petals, secretory papillae (c) can be observed by skinning, giving the surface a velvety appearance. The epidermal cells (b) have a rectangular base and appear darker than the surrounding cells. The secretory papillae are tightly packed and have a conical shape towards the outside, where anthocyanin pigments accumulate in vacuoles (a) (Figure 16).

### 3.3. Phytochemical Analysis

The levels of flavonoids, polyphenols, and anthocyanins differed between the two studied populations, most likely due to the distinct pedoclimatic conditions in which the plants developed. Overall, GH2 exhibited higher contents of polyphenolic and flavonoid compounds, as well as anthocyanins, compared to GH1, as shown in Table 3.

Statistical analysis revealed that the difference between populations was significant only for total phenolic content. GH2 (437.68 ± 9.68 mg GA/g dw) presented a significantly higher value than GH1 (315.29 ± 66.18 mg GA/g dw; *p* = 0.034). TFC was slightly higher in GH2 (27.37 ± 1.18 mg Q/g dw) compared to GH1 (25.75 ± 1.11 mg Q/g dw), but the difference was not statistically significant (*p* = 0.160). Similarly, the anthocyanin content in GH2 (4.41 ± 0.64 mg C3G/100 g dw) exceeded that of GH1 (3.55 ± 0.13 mg C3G/100 g dw), yet the difference did not reach the significance threshold (*p* = 0.086).

HPLC analysis was carried out using a high-performance liquid chromatography system equipped with a DAD detector, for quantifying individual polyphenolic compounds in the extracts of the two GH populations. A targeted analysis approach was adopted, focusing on caffeic acid and apigenin, which were selected as reference standards based on their high prevalence and biological relevance in GH. This selection allowed for precise quantification of the major phenolic constituents while optimizing analysis time and reducing resource consumption. Caffeic acid exhibited a retention time of 7.4 min with a maximum absorbance at 327 nm, while apigenin was detected at 14.4 min with a maximum absorbance at 338 nm. The calibration curves for these standards showed excellent linearity, with determination coefficients (R^2^) of 0.9990 for apigenin and 0.9994 for caffeic acid. The limits of detection (LOD) were 15.6 μg/mL for apigenin and 5.5 μg/mL for caffeic acid, while the limits of quantification (LOQ) were 26.1 μg/mL and 11.0 μg/mL, respectively, confirming the high sensitivite and precision of the method.

**Figure 17:**
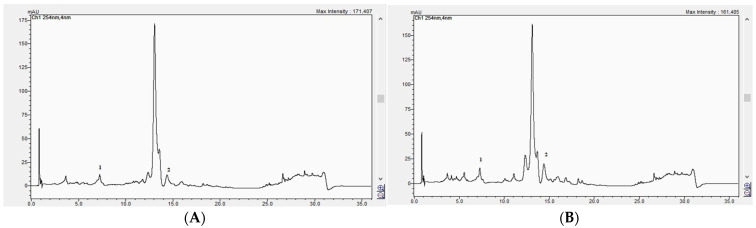
HPLC chromatograms of sample GH1 (**A**) and GH2 (**B**) recorded at 254 nm. Peaks: 1 = caffeic acid, 2 = apigenin. GH1 = plant samples collected from Săldăbagiu de Munte (261 m a.s.l.); GH2 = plant samples collected from Beiuș (553 m a.s.l.).

The established method was then applied to the analysis of the GH extracts. Figure 17A,B) presents the HPLC chromatograms of the analyzed samples, recorded at 254 nm.

The HPLC analysis revealed significant differences in the content of caffeic acid and apigenin between the two GH populations (Table 4). Caffeic acid was detected at a significantly higher concentration in GH2 (6.80 ± 0.075 mg/100 g dw) compared to GH1 (1.70 ± 0.024 mg/100 g dw; *p* < 0.001). Similarly, the apigenin content was significantly higher in GH2 (8.80 ± 0.068 mg/100 g dw) than in GH1 (6.20 ± 0.051 mg/100 g dw; *p* < 0.001). These results indicate that the pedoclimatic conditions specific to the GH2 collection site may have favored the accumulation of these phenolic compounds.

### 3.4. Antioxidants Capacity

The antioxidant capacity assessed by the CUPRAC method showed a highly significant difference between the two GH populations (*p* < 0.001). GH2 recorded a markedly higher value (1172.25 ± 10.52 μmol Trolox/g dw) compared to GH1 (398.36 ± 4.52 μmol Trolox/g dw). Similarly, the FRAP assay revealed a significant difference (*p* = 0.013), with GH2 presenting a higher reducing power (724.73 ± 9.15 μmol Trolox/g dw) than GH1 (628.15 ± 5.63 μmol Trolox/g dw). In both assays, different significance letters assigned to the same column indicate statistically significant differences (*p* < 0.05) between GH2 and GH1. These results are presented in Table 5.

## 4. Discussion

The comparative analysis of the two GH populations revealed some macroscopic and phytochemical differences, which emphasizes the importance of ecological factors in the accumulation of secondary metabolites, like the observations made by Zhou et al. and Šeremet et al. [18,25].

The macroscopic analysis reveals several morphological differences between GH1 and GH2. In GH1, the aerial stems are thinner and more elongated, and the plants generally reach greater heights compared to GH2 population. The leaves of GH1 show a lighter green hue, while in GH2 the abaxial surface of the leaf shows a darker green coloration, with violet-gray hues. This chromatic distinction is also evident in the flowers, with the corolla of GH2 showing a more intense violet pigmentation compared to that of GH1.

The microscopic characterization revealed an anatomical conformation specific to the species, with peculiarities such as the epidermis having multicellular protective trichomes and secretory glands. In cross-section on the leaf surface, both populations show multicellular secretory glands, each gland being composed of eight closely connected secretory cells. These aspects were similar in both GH populations. The distribution and density of protective trichomes on the leaves are remarkably high. Differences observed in the GH2 leaves show a higher number and longer protective trichomes compared to GH1. This suggests a possible local adaptation or ecotypic polymorphism—an observation in line with the morphological studies previously carried out on other species of the Lamiaceae family, useful for the authentication of plant products [26].

The differences observed between GH1 and GH2 populations may be interpreted as ecological adaptations. For example, the higher density and length of protective trichomes in GH2 could be related to microclimatic stress factors, including increased UV radiation and lower humidity at higher altitude. Similar correlations between trichome density and ecological factors have been described in other Lamiaceae species, such as *Mentha species* and *Salvia nemorosa* [40,41,42]. Trichomes play a crucial role in regulating transpiration, protecting against UV radiation, and serving as storage sites for volatile secondary metabolites [43,44].

The more intense violet pigmentation of GH2 petals may indicate an increased accumulation of anthocyanins, which are known to act as photoprotective pigments. Studies have demonstrated that exposure to high levels of UV-B radiation stimulates anthocyanin biosynthesis in leaves, flowers and fruits thereby enhancing plant tolerance to environmental stress [45,46,47].

Phytochemically, the differences observed between GH1 and GH2 may be interpreted as a chemotype variation. Environmental factors such as soil composition, temperature, and altitude strongly influence the accumulation of phenolic acids, flavonoids, and anthocyanins in plant species. Previous studies on GH collected from different regions of Europe have reported significant quantitative variations in phenolic derivatives and flavonoid content [22,48].

The anatomical and histological observations obtained for GH1 and GH2 confirm the authenticity of the plant material through the identification of diagnostic traits such as multicellular secretory glands and multicellular protective trichomes, which ensure that the raw material used for extraction is correctly identified and standardized, thereby contributing to the overall quality control of the resulting supplements or natural products.

Polyphenols are among the most important secondary metabolites in GH, contributing substantially to its antioxidant potential. These compounds, which include phenolic acids (such as caffeic, chlorogenic, and rosmarinic acids) and flavonoids (such as apigenin, luteolin, and rutin), act as effective free radical scavengers and metal chelators, thereby protecting plant tissues from oxidative stress. Other studies have shown a strong correlation between total polyphenol content and antioxidant capacity in GH, regardless of the extraction method employed, and have demonstrated that different treatment processes, harvest times, and cultivation environments can significantly influence its phytochemical composition, including essential oils, flavonoids, phenols, terpenoids, and organic acids [18].

The higher antioxidant capacity observed in GH2, as indicated by both CUPRAC and FRAP assays, is consistent with its greater content of phenolic compounds, including total polyphenols, flavonoids, and anthocyanins, as well as higher concentrations of individual phenolics such as caffeic acid and apigenin. The fact that GH2 originates from a higher-altitude habitat suggests that pedoclimatic conditions, such as increased UV radiation, temperature fluctuations, and other abiotic stresses, may stimulate the biosynthesis of phenolic metabolites with antioxidant properties. This combination of a richer phytochemical profile and greater antioxidant activity supports the potential of GH2 as a superior source of bioactive compounds for pharmaceutical and nutraceutical applications. The results of the study emphasize the phytochemical characteristics of the species GH, supported by histo-anatomical observations and the identification of phenolic compounds by HPLC analysis. The comparative analysis of the two populations GH1 and GH2 revealed important variations in the content of bioactive compounds (caffeic acid, apigenin), which underlines the influence of ecological conditions on the biosynthesis of secondary metabolites, as reported in other recent studies [18,22,25].

HPLC analysis confirmed the presence of phenolic compounds such as apigenin and caffeic acid, which have previously been reported among the major constituents of GH extracts by Chou et al. and Sławińska et al. [21,22]. Apigenin, a flavonoid recognized for its antioxidant, anti-inflammatory, and potentially anticancer activity, was present in increased concentrations in GH2 (8.80 ± 0.068 mg/g) compared to GH1 (6.20 ± 0.051 mg/g), suggesting a possible increased pharmaceutical relevance of this population.

Previous studies on pharmaceutically important species of the Lamiaceae family have demonstrated that phenolic acids (e.g., caffeic acid) and flavonoids (e.g., apigenin) are widely distributed and play a major role in their antioxidant properties. For example, *Mentha* species have been reported to contain caffeic and rosmarinic acids, together with flavonoids such as luteolin and apigenin [49,50].

Likewise, both *Rosmarinus officinalis* (rosemary) [51] and *Salvia species* (sage) [52] contain caffeic acid, apigenin, and rosmarinic acid, together with other phenolic compounds, all of which contribute to their antioxidant activity.

In this context, our findings of caffeic acid and apigenin in GH align with the phytochemical profiles described for other pharmaceutically relevant Lamiaceae species.

One of the main factors determining the differential accumulation of secondary metabolites in GH is soil composition. Altitudinal variation is closely reflected in soil characteristics: at 261 m a.s.l. (for GH1), soils are more fertile and support vigorous vegetative growth, while at higher altitudes, such as 553 m a.s.l. (for GH2), soils tend to be more acidic, poor in nutrients, and exhibit variable water retention capacity. These conditions impose an ecological stress that favors the accumulation of bioactive compounds, especially phenolic acids, flavonoids, and anthocyanins. This observation supports the hypothesis that the GH2 population has undergone an adaptation to specific ecological constraints.

Overall, the comparative analysis of the GH1 and GH2 populations highlights both morphological and phytochemical variability, providing evidence of ecological adaptation and chemotype differentiation. These findings reinforce the relevance of our study for understanding the link between plant biodiversity, environmental stressors and pharmacognostic potential.

Current limitations include the lack of in vivo validations and pharmacokinetic testing, which will be addressed in future research. Therefore, morpho-anatomical, microscopic and phytochemical characterization can constitute an essential step in the process of standardization and authentication of plant material, thus facilitating the integration of GH extracts in clinical or nutritional applications. In addition, the chromatographic method applied did not achieve complete resolution for all polyphenols, as minor interfering peaks were observed and the resolution of apigenin remained limited. Moreover, rosmarinic acid (a predominant phenolic constituent with strong antioxidant relevance in *Lamiaceae species*) was not included in the present analysis. These aspects may partially restrict the interpretation of our phytochemical findings and should be further addressed in future investigations.

## 5. Conclusions

This paper provides cumulative, relevant and authentic information on GH from the spontaneous flora of Bihor County, a region in north-western Romania. To our knowledge, no previous studies have examined GH from these habitats in terms of its botanical, phytochemical, and antioxidant characteristics. The botanical, morphological and anatomical characteristics of both vegetative and reproductive organs of the GH have now been described for the first time, representing important data for the correct identification of the species. Both GH1 and GH2 have some morphological changes (shorter stems, more intense color of leaves and flowers, a larger number of protective trichomes at higher altitudes), such as ecological adaptation, in response to environmental conditions (altitude, soil, temperature, humidity, solar radiation, etc.). However, phytochemical and HPLC analysis revealed differences in the concentrations of apigenin and caffeic acid across the two analyzed populations. Our results provide new insights into the morphological, anatomical, and phytochemical variability of GH, supporting its potential as a natural source of bioactive compounds with antioxidant properties. In this context, it is essential to fully understand the botanical, morphological, anatomical and phytochemical characteristics specific to a plant species in order to ensure accurate identification and to avoid undesirable substitutions.

## Figures and Tables

**Table 1:** Annual pedoclimatic and ecological characteristics of the two studied regions.

Parameter	GH1—*Glechoma hederacea* L., Săldăbagiu de Munte	GH2—*Glechoma hederacea* L., Beiuș
Geographical setting	Hilly area (Crișana piedmont) alt. 261 m	Mountain area near Beiuș Depression (Apuseni Mountains) alt. 553 m
Mean annual temperature	10.5–11 °C	8–9 °C
Mean annual precipitation	600–650 mm	800–1000 mm
Soils	Luvisols, Rendzinas; lighter, well-drained	Cambisols, Luvisols, Rendzinas; higher organic matter, more moisture
Light exposure	Higher, open relief, south-facing slopes	Reduced by mountain relief and forest cover
UV radiation	Higher due to slope orientation and open landscape	Moderated by cloud cover and orographic shading
Ecological conditions	Agricultural land, vineyards, xerophilous grasslands	Beech and mixed forests, natural grasslands, humid habitats

**Table 2:** Morphological characteristics of *Glechoma hederacea* L. populations (GH1 vs. GH2).

Characteristic	GH1	GH2
Plant height (cm)	21.5 ± 0.5	10.5 ± 0.5
Stem thickness (mm)	6.5 ± 0.5	5.5 ± 0.5
Petiole length (cm)	4.5 ± 0.5 (longer than blade)	2.2 ± 0.2 (equal to blade)
Leaf blade length (cm)	~3.0 ± 0.2	~2.2 ± 0.2
Leaf shape	reniform, slightly elongated	reniform, less elongated
Leaf color (adaxial)	dark green	dark green
Leaf color (abaxial)	light grey-green	brown with purple shades
Leaf vein color	palmate, green veins	palmate, light brown veins
Stem color	light green	green with purple tinges
Petal color	light purple	dark purple
Sepal color	green	green with brown-purple tinges
Protective trichome density (stem, per cm^2^)	lower	higher
Protective trichome morphology (stem/petiole)	shorter, thinner	longer, thicker
Protective trichome morphology (petal)	thicker, rounded tip	thinner, pointed tip
Petal pigmentation	upper petals uniformly purple; lower with darker spots	upper/lower petals darker purple, more intense

**Table 3:** Content of bioactive compounds in the two populations of *Glechoma hederacea* L. and statistical significance of differences.

Bioactive Compounds	GH1	GH2	*p*-Value
Total phenolic content (mg GA/g dw)	315.29 ± 66.18 ^a^	437.68 ± 9.68 ^b^	0.034
Flavonoids content (mg Q/g dw)	25.75 ± 1.11 ^a^	27.37 ± 1.18 ^a^	0.160
Total anthocyanin content (mg C3G/100 g dw)	3.55 ± 0.13 ^a^	4.41 ± 0.64 ^a^	0.086

Values are expressed as mean ± standard deviation (SD), *n* = 3. GH1 = plant samples collected from Săldăbagiu de Munte (261 m a.s.l.); GH2 = plant samples collected from Beiuș (553 m a.s.l.). GA = gallic acid; Q = quercetin; C3G = cyanidin-3-glucoside. Values with different letters in the same row are significantly different according to the independent samples *t*-test (*p* < 0.05).

**Table 4:** The identification and quantification data obtained by HPLC.

Sample	Compound	Subclass	R_t_ (min)	UV λ_max_ (nm)	Quantification (mg/100 g dw)
GH1	Caffeic acid	Hydroxycinnamic acid	7.6	327	1.70 ± 0.024 ^a^
	Apigenin	Flavone	14.4	340	6.20 ± 0.05 ^A^
GH2	Caffeic acid	Hydroxycinnamic acid	7.3	327	6.80 ± 0.075 ^b^
	Apigenin	Flavone	14.4	339	8.80 ± 0.068 ^B^

Values represent mean ± standard deviation (SD) of three replicates (*n* = 3). Different letters indicate statistically significant differences between GH1 and GH2 for each compound (*p* < 0.05). GH1—plant samples collected from Săldăbagiu de Munte (261 m a.s.l.); GH2—plant samples collected from Beiuș (553 m a.s.l.).

**Table 5:** Antioxidant activity determined by the two chemical methods.

Sample	CUPRAC	FRAP
	µmol TE/g dw	
GH1	398.36 ± 4.52 ^b^	628.15 ± 5.63 ^b^
GH2	1172.25 ± 10.52 ^a^	724.73 ± 9.15 ^a^

Values represent mean ± standard deviation (SD) of three replicates (*n* = 3). Different letters within the same column indicate statistically significant differences between GH1 and GH2 (Student’s *t*-test, *p* < 0.05). GH1—plant samples collected from Săldăbagiu de Munte (261 m a.s.l.); GH2—plant samples collected from Beiuș (553 m a.s.l.).

## Data Availability

The original contributions presented in the study are included in the article. Further inquiries can be directed to the corresponding author.

## Abbreviations

The following abbreviations are used in this manuscript:

A	Absorbance
AlCl_3_	Aluminum chloride
CUPRAC	Cupric ion reducing antioxidant capacity
DAD	Diode array detection
DF	Dilution factor
DW	Dry weight
FeCl_3_	Ferric chloride
FRAP	Ferric reducing antioxidant power
GA	Gallic acid
GH	*Glechoma hederacea* L.
GH1	*Glechoma hederacea* L., population 1 from Săldăbagiu de Munte (Bihor County) (47°5′22″ N 21°58′31″ E) altitude 261 m
GH2	*Glechoma hederacea* L., population 2 from Beius (Bihor County) (46°34′47″ N 22°12′59″ E) altitude 553 m
HCl	Hydrochloric acid
HPLC	High performance liquid chromatography
MW	Molecular mass
Na_2_CO_3_	Sodium carbonate
NaNO_2_	Sodium nitrite
NaOH	Sodium hydroxide
Q	Quercetin
SD	Standard deviation
TFC	Total flavonoids
TPC	Total phenolic content
TPTZ	Tripyridyl triazine complex
UV-VIS	Ultraviolet–visible
ε	Molar extinction coefficient
